# Drivers of farmers’ intentions to adopt genome-edited cassava resistant to cassava brown streak disease in Uganda

**DOI:** 10.1080/21645698.2026.2698267

**Published:** 2026-07-08

**Authors:** Alice Onek Atimango, Stephen Wamala Kalule, Brian Senyange, Wim Verbeke, Hans De Steur

**Affiliations:** a Department of Agricultural Economics. Faculty of Bioscience Engineering, Ghent University, Ghent, Belgium; b Department of Rural Development and Agribusiness. Faculty of Agriculture and Environment, Gulu University, Gulu, Uganda

**Keywords:** Adoption intentions, affective and cognitive drivers, cassava, farmers, genome editing, structural equation modeling, Uganda

## Abstract

Genome editing has been increasingly promoted as a promising breeding technology to strengthen the agrifood system. However, its success largely depends on farmers’ adoption of the technology, a topic that remains insufficiently addressed in current literature. This study applies the model of goal-directed behavior (MGB) to examine farmers’ adoption intention for genome edited (GE) cassava, resistant to cassava brown streak disease, in a hypothetical scenario in a developing country context. Data was collected from a cross-sectional survey of 357 cassava farmers in Northern Uganda and analyzed using partial least squares structural equation modeling. The results show that farmers have positive intentions toward adopting GE cassava if it becomes available in the market. However, their intentions to adopt depend on their attitude, perceived behavioral control, desire, positive anticipated emotions and subjective norms. Negative anticipated emotions and subjective norms had no significant influence on the desire to adopt GE cassava. The findings also highlight the mediating role of desire and the role of affective drivers in shaping adoption intentions, underscoring the importance of dual process perspectives in agricultural technology adoption. Farmers’ education level and experience emerged as important moderators for certain model pathways, revealing a need for segmented approaches for fostering the adoption of GE cassava once they are available. This study contributes empirically by extending the application of the MGB to agricultural biotechnology and offers practical insights for future development and deployment of GE crops in developing country contexts like Uganda.

## Introduction

Genetic engineering technologies have often been put forward for their great potential to enhance the resilience and sustainability of the agrifood system.^1–3^ However, technologies like genetic modification have not received very positive reactions from the global public. While various regions of the world slowly embraced the technology, evident with an increase in acreage under genetically modified (GM) crops cultivation,^4^ the adoption of GM crops in Africa remains slow. This is due in part to lack of information, misinformation on health and safety of GM foods, negative public opinions toward biotechnology, and regulatory hurdles imposed on GMOs.^5–7^

Beyond these broad constraints, a substantial body of literature on agricultural technology adoption in developing countries highlights that farmers’ decisions are shaped by a complex interplay of economic, institutional, and socio-cultural factors.^8,9^ Previous studies emphasize the importance of performance expectancy,^10^ anticipated benefits and risks,^11^ and prevailing market conditions.^12^ Empirical evidence from GM crop adoption, particularly Bt cotton and maize in countries such as South Africa and India suggests that farmers are generally willing to adopt technologies that reduce production risks and improve yields, but adoption outcomes vary considerably depending on local agro-ecological conditions, input availability, and farm-level characteristics.^13,14^ For instance, Nazia^13^ found that South African commercial farmers derive greater benefits from using GM crops compared to small holder farmers, who often remain marginalized. Furthermore, unequal access to certified seeds, information asymmetries, and weak extension systems continue to constrain adoption, even when economic benefits are evident.^8,15^ These findings underscore that technological superiority alone may be insufficient to guarantee adoption, but rather a complex interplay of several factors matters.

Similarly, studies on improved seed technologies, including drought-tolerant maize and disease-resistant cassava, demonstrate that adoption decisions are highly context-specific, and strongly influenced by farmers’ access to seeds, information and perceptions of varietal attributes.^15,16^ For instance, Mondo et al.^17^ report that despite the agronomic advantages of improved cassava varieties in the Democratic Republic of Congo, including enhanced yield potential, disease resistance and early maturity, adoption remained limited because these varieties did not match well with local farmer’s preferences related to leaf production, in-soil storage and tuber color. As a result, farmers continued to favor traditional landraces having these desirable attributes. This underscores the need for more participatory and farmer-centered innovation approaches, recognizing that alignment between technological attributes and local livelihood needs is critical for successful uptake,^18^ especially in contexts where consumption habits and cultural preferences play a central role in varietal acceptance.

Contrary to earlier biotechnologies, new breeding techniques (NBTs) like genome editing (GE) have received more positive perceptions from the public.^19,20^ CRISPR genome editing allows for targeted manipulation of the genome of an organism in a simple, precise, and accurate manner, thus enabling faster breeding for developing improved crop varieties.^21^ Previous research has lauded the technology for its potential in improving crop yield, enhancing resilience to climate change (e.g. high temperatures and water scarcity),^22,23^ boosting resistance to biotic stresses such as pests, diseases and weeds,^24^ leading to reduced chemical use in the agrifood system, and consequently limiting the impact of agriculture on the environment. Post harvest, the technology has also been reported to reduce food loss and waste through elongation of the shelf life of fresh produce, and improve nutritional and sensory quality of food.^25,26^ Hence, attributable to its contributions to the food system, GE could contribute to achievement of several of the sustainable development goals (SDGs) and, in particular, to Africa’s Agenda 2063.

Despite these advantages, empirical evidence on how farmers, especially in developing countries, perceive and evaluate GE remains limited. Studies suggest that farmers and other stakeholders may distinguish GE from GM technologies, particularly where regulatory frameworks and communication strategies emphasize its precision and relevance to local challenges.^27^ Nonetheless, adoption dynamics are still likely to reflect broader patterns observed in earlier agricultural innovations, where factors such as institutional trust, perceived risks and benefits, and compatibility with existing production systems play a decisive role.

Importantly, previous research has consistently shown that the adoption and success of agricultural technologies and innovations are greatly influenced by multiple stakeholders across the food supply chain.^28^ Moreover, it is important to recognize that while technology may bring profound changes within a society, its ultimate success and adoption are determined by the society in which it is implemented.^29^ To this effect, a growing number of studies have examined stakeholder perspectives toward GE in food and agriculture. However, most of these studies have focused on consumers,^30–34^ particularly in developed countries. So far, relatively little attention has been paid to the perspectives of farmers as primary food producers,^28,35,36^ and empirical evidence specifically focussing on crop farmers remains especially scarce. One of the few crop-focused studies^37^ found that Italian farmers held generally positive attitudes toward CRISPR/Cas blast resistant rice but were skeptical of the potential agribusiness risks associated with its adoption. Beyond this, most available evidence on farmers’ evaluation of GE stems from the livestock sector, offering indicative insights into how farmers assess NBTs under conditions of uncertainty. For example, while profitability is an obvious consideration prior to adoption of a new technology,^38^ farmers in the United States did not view profit alone as sufficient motivation for adopting GE cattle.^39^ Instead, acceptance of GE was significantly influenced by peer adoption, recommendations of veterinarians, attribute variables of GE, and farm/farmer characteristics. Similarly, Danish dairy farmers were more inclined to accept GE if the breeding goal aimed to improve animal welfare or reduce the impact of climate change, rather than increase milk quantity.^40^ Meanwhile, US farmers were reported to have a positive attitude toward GE if it would provide them with economic benefits, though they remain skeptical of the technology’s naturalness and potential risks.^41^ Although these studies provide valuable insights, they predominantly reflect perspectives from developed countries and from livestock rather than crop production sector, thereby overlooking the contextual differences that may shape perceptions and adoption patterns among crop producers from developing regions.

Despite the growing scholarly interest in stakeholder evaluation of GE in agriculture, a significant empirical gap still remains regarding perceptions of farmers in developing regions, particularly in Africa where small holders dominate food production. Evidently, socioeconomic studies have concentrated on consumers, leaving limited understanding of farmers’ motivations, intentions and concerns regarding GE. This oversight is critical, as farmers are pivotal in the success of agricultural innovations, and their decisions to adopt the cultivation of GE crops determine whether other chain actors like consumers can access them. Thus, sidelining farmers from GE research and discourse risks marginalizing a key stakeholder group, giving room for other interest groups to dominate the narrative on their behalf, as was observed with the GMO debate.^42^

To address this gap, this study adopts the Model of Goal-Directed Behavior (MGB)^43^ to analyze farmers’ adoption intentions in the context of GE. This framework extends traditional behavioral models by integrating cognitive, affective, and motivational drivers of decision-making. In addition to rational factors such as attitudes, perceived behavioral control, and social norms, the MGB explicitly incorporates anticipated emotions and desire, recognizing that farmers’ adoption intentions are not only shaped by rational and logical assessments but also by emotional responses and goal-oriented motivations. This integrative approach is particularly relevant for GE technologies, where adoption decisions may be associated with uncertainty, perceived risks, and aspirations related to productivity and livelihood outcomes, as observed with previously introduced biotechnologies.

Empirically, this study examines Ugandan farmers’ intentions to adopt GE cassava resistant to cassava brown streak disease (CBSD) in a hypothetical context. Cassava is one of the ten commodities prioritized by Uganda’s National Development Plan III (NDP III) because of its significant contribution to food and nutrition security, and for its multi-industrial use.^44^ The crop is predominantly cultivated and consumed in the Eastern (37%), Northern (34%), Western (15%) and Central (14%) regions of Uganda,^45^ serving both as a staple and vital source of income for over 70% of the value chain actors. More recently, cassava has also gained prominence in the countries’ industrial sector for its applications in baking, pharmaceuticals and breweries.^46^ Though mostly utilized domestically, cassava also contributes to Uganda’s export earnings, mainly exported in form of starch, chips or as fresh cassava to neighboring countries, including South Sudan, Rwanda and Democratic Republic of Congo.^45,47^ Nonetheless, CBSD remains a major constraint to cassava production, leading to yield loss of up to 70–100%, depending on the varietal susceptibility and viral pressure.^48–50^ Recently, scientists in Uganda embarked on developing GE CBSD-resistant cassava varieties, which could offer hope for improving cassava production in the country.^51^ While these varieties are not yet commercialized for use by farmers, their future success will still depend on farmers’ willingness to adopt them.

Accordingly, this study undertakes an ex-ante assessment of farmers’ behavioral intentions toward adopting GE cassava in a hypothetical context. Such diagnostic research is necessary for predicting potential adoption responses and for guiding evidence-based breeding and policy decisions before commercialization.^52^ Unlike earlier retrospective studies that investigated user acceptance of emerging technologies after their rejection,^53^ this study provides an early diagnostic perspective. Theoretically, this study is novel in applying the model of Goal directed behavior (MGB) to an agricultural biotechnology context, integrating both affective and cognitive drivers of adoption intentions among African farmers. Understanding these intentions and the factors shaping them is crucial for guiding both policy and practice. Empirically, and to the best of our knowledge, this is the first study to assess farmers adoption intentions for GE crops in sub-Saharan Africa. It contributes to the growing literature on evaluations of GE in agriculture, by foregrounding producers’ perspectives and situating African farmers’ opinions within the broader global discussions on genome editing in agriculture.

### Theoretical Framework and Hypotheses

Several theoretical models have been suggested and used to assess determinants of adoption intentions and behavior toward novel technologies in agriculture. The Theory of Planned Behavior (TPB) has been widely used to analyze farmers’ adoption intentions toward novel crops and agricultural technologies.^54–57^ As a consequence of its roots in the Theory of Reasoned Action (TRA), the TPB framework is skewed toward the cognitive determinants of behavior, namely attitude, perceived behavioral control and subjective norms. It posits that these three factors directly and positively influence behavioral intentions of an individual.^58^ However, the theory does not take into account the affective and emotional factors that have been recognized in previous studies as reliable predictors of human behavior,^59,60^ including farmers.^61,62^ This limitation is particularly relevant in the context of innovative and controversial technologies, where uncertainty, perceived risk, and emotional reactions may play a central role in shaping decision making.^63^ To address this limitation of the TPB, this present study adopts the model of goal directed behavior (MGB)^43^ as a more comprehensive theoretical lens to examine farmers’ adoption intentions toward GE CBSD-resistant cassava in Uganda.

The MGB extends the TPB by incorporating the emotional, motivational and the habitual factors into the decision-making process of farmers toward novel agricultural technologies such as GE. In addition to the core TPB constructs, the MGB recognizes emotions as an important factor for behavioral decision making, acknowledging that individual’s emotional experiences contribute in shaping their responses to technology,^43,60^ an aspect that is often overlooked in behavioral models. Moreover, given the controversy and scrutiny surrounding biotechnology in agriculture,^64^ emotions could be important predictors of farmers’ behavior toward the technology. Furthermore, the MGB introduces desire as a proximal determinant for intention to perform a behavior.^43^ Desire reflects the motivational drive or bridge through which both cognitive evaluations (attitude, subjective norms and perceived behavioral control) and affective responses (anticipated emotions) jointly influence behavioral intentions. Because of its motivational nature, desire is hypothesized to be the strongest predictor of behavioral intentions in the MGB.^43^ By explicitly incorporating desire as a construct, the MGB goes beyond purely cognitive evaluations and acknowledges the role of goal-oriented motivation. This emphasis on desire enables the model to capture goal orientation and motivational commitment more effectively than frameworks that assume intention is formed directly from beliefs and evaluations alone.^43,60^

The MGB has been widely applied in different research fields and contexts to better understand the intentional, motivational and emotionally driven aspects of behavior. For instance, to study health related intentions like cessation of smoking,^65^ moderate drinking,^66^ participation in sports,^67^ and eating vegetables and fruits^68^; virtual shopping intention among Canadian consumers^69^; youth traveler’s food purchase decision making at night markets in China,^70^ and tourist’s travel and revisit intentions.^71,72^ In agriculture, the MGB has been scantily employed to predict adoption intentions for internet of things (IoT) and smartphone use among farmers.^73,74^ However, to date, no empirical study has employed the MGB to examine farmers’ adoption intentions toward biotech crops or GE crops specifically. Furthermore, while emotional factors have been recognized as important predictors of farmers’ behavior in climate adaptation^75,76^ and herd management,^77,78^ no study has attempted to link farmers’ emotions as a predictor to adoption intentions in the biotechnology domain. To address these research gaps, this study contributes both theoretically and empirically to existing literature on farmers’ adoption of GE crops, by employing MGB as a comprehensive theoretical model to examine the predictors of farmers’ adoption intentions toward GE crops, with a focus on CBSD-resistant cassava in Uganda. Consequently, this study answers the following research question: What are the determinants of farmers’ adoption intentions toward GE cassava? To address this question, this study specifically examines 1) the influence of affective (emotional) and cognitive determinants on adoption intentions, and 2) the extent to which desire mediates the relationship between emotions, attitude, perceived behavioral control, and subjective norms on adoption intentions.

#### Research Hypotheses

In the MGB, emotions are considered a psychological construct independent of attitude.^43^ An individual who perceives a technology to be a threat is likely to experience different emotions from another who perceives it to be an opportunity,^79^ hence yielding negative and positive anticipated emotions, respectively. These anticipated emotions, also referred to as pre-factual appraisals, demonstrate the decision maker’s imagined consequences before engaging with novel technologies, since they directly relate to the personal goals and are associated with performing the behavior in question.^80^

Positive anticipated emotions (PAE) are favorable feelings an individual expects to experience as a result of performing a behavior, such as satisfaction, happiness, pride or excitement.^81^ In contrast, negative anticipated emotions (NAE) are unfavorable expectations, such as disappointment, sadness, failure and frustration.^82,83^ Individuals anticipating negative emotions are generally less motivated to perform a behavior due to their expectation of an undesirable outcome.^84^ Studies have demonstrated the crucial role of anticipated emotions in influencing individual’s decision and the motivation to perform a behavior. For instance, PAE (pride) were reported to have influence on farmers’ adaptive response to climate change.^75^ Negative emotions (anxiety) were also associated with consumer acceptance of crops produced with new breeding technologies in Japan.^85^ Emotions have also been associated with farmers intentions to adopt smartphones and IoT sensors for agricultural use, both directly and indirectly through desire.^73,74^ In the context of this study, the possibility of adopting GE cassava is likely to evoke positive emotions if the anticipated benefits outweigh the risks, whereas negative emotions may arise if harm is anticipated. Consistent with the MGB, the influence of both emotions on behavioral intentions is expected to be mediated by desire (DE) to perform the behavior.^43^ Thus, we hypothesize that PAE and NAE are direct predictors of desire.

H1: Positive anticipated emotions have a positive effect on desire.

H2: Negative anticipated emotions have negative effect on desire.

Consistent with the TPB, attitude (ATT), subjective norms (SN) and perceived behavioral control (PBC) are also included in the MGB used in this study. Attitude refers to an individual’s evaluation of a particular behavior in terms of its positive or negative attributes.^84^ Subjective norms refer to the perceived social pressure to engage in or refrain from a behavior based on the opinions of the people an individual considers important.^58^ Perceived behavioral control is an individual’s perception of his confidence or ability to perform a particular behavior.^86^ Previous studies employing the MGB have posited that SN, PBC and ATT have an indirect influence on intention, with their influence mediated by an individual’s desire to perform the behavior.^43,66,73,87^ Moreover, these three constructs have been reported to significantly explain more variance in desire than in intentions.^88^ However in the TPB model, PBC, ATT and SN have also been shown to have a direct effect on the intention to perform a behavior.^54,89^ Therefore, we hypothesize:

H3a: Attitude has a positive influence on desire.

H3b: Attitude has a positive influence on adoption intentions.

H4a: Subjective norms has a positive influence on desire.

H4b: Subjective norms has a positive influence on adoption intentions.

H5a: Perceived behavioral control has a positive effect on desire.

H5b: Perceived behavioral control has a positive effect on adoption intentions.

Desire in the MGB is an individual’s motivation to engage in a behavior or achieve a goal.^90^ Desire plays a key role as a motivational factor in decision making,^91^ not only as a direct predictor of behavioral intention but also as a mediating construct through which other antecedents including attitude, subjective norms, PBC and anticipated emotions influence behavioral intentions.^43^ Therefore, the following hypothesis is proposed:

H6: Desire has a positive effect on adoption intentions.

**Figure 1.**
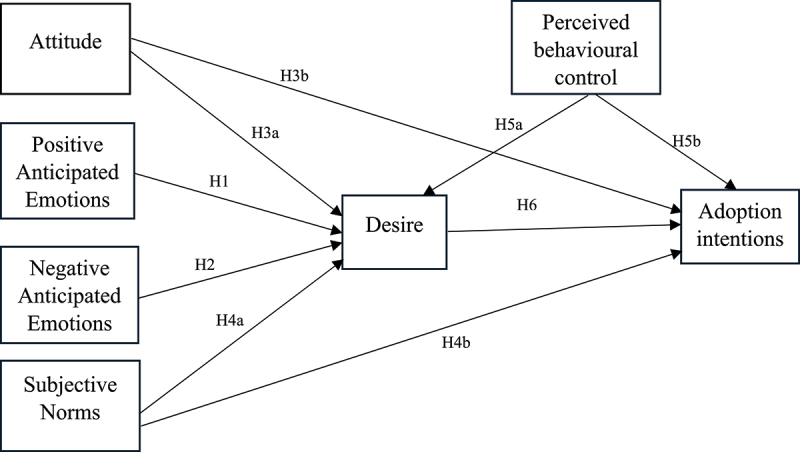
Hypothesised model for adoption intentions towards GE cassava. Authors own elaboration based on the TPB^58^ and MGB^43^.

All hypothesized relationships tested in this study are visually presented in the conceptual framework in Figure 1.

Group comparisons have been used in previous studies to show variations within population subgroups.^92^ Typical of socioeconomic studies like the present one, where there is marked variation within the sampled population, there is a need to assess this variability without generalizing the results to prevent drawing significantly skewed conclusions.^93^ Specific to the literature on GE in agriculture, several studies have revealed that sociodemographic factors including gender, age, income status, education, farming experience and experience with biotechnology crops have varying impacts on perceptions, adoption intentions (AI) and general evaluations of such crops and foods.^39,40,94–97^ Although sociodemographic variables were not incorporated into the proposed model, their effects were examined in the subsequent subgroup analysis to identify any potential differences in the model pathways. Acknowledging these differences offers valuable insights for scientists, extension service providers, policymakers and science communicators to develop tailored communication strategies that can address the information needs of the diverse farmer groups.

## Materials and Methods

### Study Design

A quantitative cross-sectional, questionnaire-based survey was conducted with 357 cassava farmers in Gulu district of Northern Uganda, selected using a non-probabilistic purposive sampling approach. The questionnaire was developed and pre-tested with 22 farmers, after which adjustments were made on the wordings of some of the items. The survey consisted of two parts. The first part captured the sociodemographic variables of the respondents including gender, age, education level, farm size, annual farm income, farm area under cassava cultivation and experience with improved crop varieties. In the context of this study, improved varieties relate to all crop varieties that are bred or selected to perform better than the local or traditional varieties under specific conditions. These improved varieties often offer higher yields and greater resilience to biotic and abiotic stresses.^98^ This explanation was provided to the respondents using existing commercialized, and well known varieties before asking this particular question.

The second part examined the constructs of the MGB to measure farmers’ AI for the genome edited cassava. To give a background on GE technology, a short information script describing GE technology and its use in agriculture was given to the respondents (see Supplementary material S1). The information script was adapted from Baum et al^99^ and co-designed in consultation with 21 individuals including biotechnologists, university professors, researchers and agricultural extension officers, to ensure that the information is correct, balanced and simplified enough to be understood by the respondents. The script contained both the benefits of GE and the uncertainties that are still being questioned, offering a balanced and simplified information for the respondents. The seven constructs of the MGB, including ATT, SN, PAE, NAE, DE, PBC and AI, were all assessed using multiple items adopted from previous literature. Accordingly, ATT was measured using five items,^43,100^ SN was measured using four items,^74,100^ PBC was measured using three items,^100^ PAE was measured using three items,^43,73^ NAE had three items.^43,73,74^ DE was measured using two items^43,73,74^ and AI was measured using three items.^76,100^ All items were measured on a 5-point Likert scale ranging from 1 (strongly disagree) to 5 (strongly agree). Details of the constructs and measurement items are provided in Table 1.

**Table 1.** Constructs and measurement items, with respective descriptive statistics (mean and standard deviation SD on 5-point likert scale, *n* = 357).

Constructs	Items	Mean	SD
**Attitude**			
ATT1	It would be a good idea for me to cultivate GE cassava	4.69	0.60
ATT2	Adopting GE cassava would enhance my farm’s productivity	4.45	0.62
ATT3	I do not like the idea of cultivating GE cassava on my farm	4.76	0.57
ATT4	I am positive towards cultivating GE cassava on my farm	4.47	0.62
ATT5	Growing GE cassava could be beneficial for my farm	4.49	0.61
**Subjective Norms**			
SN1	People who are important to me would adopt GE cassava	4.52	0.59
SN2	Religious leaders and cultural leaders do not support my decision to grow GE cassava	4.38	0.78
SN3	People who are important to me would think that I should cultivate GE cassava	4.65	0.72
SN4	My fellow farmers would support the use of GE cassava	4.45	0.60
**Perceived Behavioural Control**			
PBC1	I am confident in my ability to grow GE cassava if made available	4.54	0.60
PBC2	If I want to, I would grow GE cassava	4.50	0.94
PBC3	It is up to me to decide whether to cultivate GE cassava on my farm or not	4.43	0.73
**Positive Anticipated Emotions**			
PAE1	I would feel proud if I successfully grow GE cassava	4.71	0.45
PAE2	I would feel satisfied if I successfully grow GE cassava	4.60	0.53
PAE3	I would feel happy if GE cassava performs well in my garden	4.76	0.62
**Negative Anticipated Emotions**			
NAE1	I would feel angry if GE cassava does not meet my expectation	4.25	0.95
NAE2	I would feel disappointed if GE cassava does not meet my expectation	4.11	0.94
NAE3	I would feel sad if GE cassava does not meet my expectation	4.48	1.00
**Desire**			
DE1	I desire to grow GE cassava if it is available	4.60	0.55
DE2	I want to cultivate GE cassava to improve cassava production on my farm	4.52	0.65
**Adoption intentions**			
AI1	I plan to acquire GE cassava when they become available	4.56	0.59
AI2	I will make an effort to integrate GE cassava into my farming practices	4.55	0.58
AI3	I intend to grow GE cassava as soon as it becomes available	4.59	0.58

Note: ATT = attitude, NAE = negative anticipated emotions, DE = desire, PAE = positive anticipated emotions, PBC = perceived behavioural control, SN = subjective norms, AI = adoption intentions.

### Data Collection

Data was collected using face-to-face interviews with selected cassava farmers in Gulu district, Northern Uganda in July 2025. Participation was voluntary and based on respondents’ informed consent. Trained research assistants administered the questionnaires to respondents using mWater.^101^ This application can be used offline, making it suitable for data collection in rural areas, the typical context for this study.

As of 2026, Uganda continues to rely on the Uganda National Council for Science and Technology (UNCST) Act of 1990 to regulate all research in the country, including that on genetic engineering.^102^ The country has not yet commercialized/cleared GMOs for cultivation in farmers’ fields. Despite Uganda’s national biotechnology strategy recognizing biotechnology as a tool to address issues in the agricultural sector, and the hefty investment in functional biotechnology research facilities, regulatory hurdles remain a challenge. Consequently, little attention has been given to genome editing in Uganda in terms of regulations, media discussions and socioeconomic research. Until very recently, the drafting of the CRISPR regulation/guidelines has started to guide the development and implementation of GE in the country.^103^ While research in GE crops is ongoing in the research institutes, no field trials have been established yet.^102^ Scientists in the country are using GE to develop a cassava variety that is resistant to the CBSD,^51^ and induce early flowering in cassava,^104^ offering significant strides toward enhancing the crop’s productivity and resilience to stresses.

Gulu district was chosen as study area because of its high reliance on cassava as a food crop. In the past years, the region has reported lower yield performances of cassava, subsequently affecting both income and food security of the farmers.^105^ Additionally, several projects have in the past promoted high yielding and disease resistant cassava varieties in the area as a major food security crop, post recovery from the 2-decade insurgency that the region suffered. Moreover, Gulu is also one of the districts that have had a high prevalence of the CBSD (47.1%) since its reemergence in the country in 2005.^49,106^

### Data Analysis

Data was imported into SPSS 31 and screened for outliers and missing data. Reverse-coding was done for the negatively worded items in the respective constructs. Descriptive statistics including means, frequencies and standard deviation were computed to characterize the respondents.

Partial Least Squares Structural Equation Modeling (PLS-SEM) was performed in SMART-PLS 4 to examine the predictive relationship between the constructs of the MGB and to test the proposed hypotheses.^107^ PLS-SEM was deemed methodologically fit for this study because of its predictive orientation and suitability in handling complex model structures with multiple constructs and indicators.^107,108^ Furthermore, the PLS-SEM automatically employs the central limit theorem to transform non-normal data that is typical of social science studies like this one.^109^

A reflective measurement model was hence defined and evaluated to describe the relationship between the latent variables and their respective indicators. Both the structural and measurement models were evaluated for model fit and quality. Assessing the measurement model included an evaluation of the reliability and validity of the model constructs, while the structural model focussed on the relationship between the constructs.^110^ The measurement model was assessed using the Cronbach’s alpha and composite reliability^108^ for construct reliability, average variance extracted (AVE) for convergent validity,^107^ and heterotrait-monotrait (HTMT) criterion for the discriminant validity.^111,112^ The structural model was assessed using several indices including the path coefficients, the variance inflation factors (VIF), effect sizes (f^2^), predictive relevance (Q^2^), and the predictive accuracy (adjusted R^2^).^107^ A bootstrapping procedure of 5000 sample draws was applied to test the proposed hypotheses at the 0.05 significance level.

Finally, to assess whether structural relationships varied across farmers’ sociodemographic groups, a PLS multigroup analysis (PLS-MGA) was conducted.^107^ Prior to this, measurement invariance was evaluated using the three-step Measurement Invariance of Composite Models (MICOM) procedure to establish configural and compositional invariances, as well as equality of mean and variance across groups.^113^ In line with PLS-MGA methodological recommendations, only variables that met the first two MICOM criteria were retained for PLS-MGA. Accordingly, age, education, income, farming experience and farm size were included, while the rest of the sociodemographic variables were excluded. The included variables were then recoded into dummy variables for the PLS-MGA analysis.

## Results

### Sociodemographic Characteristics of Respondents

Table 2 shows the sociodemographic profile of the respondents. The sample consisted mostly of female farmers (61.3%), with a mean age of 42.3 y and an average household size of 6.29 persons. More than half of the respondents had attained primary education (66.4%) and the remaining secondary education (19.3%), other institutions (vocational/tertiary) of learning (3.9%) or no formal education at all (10.4%). No single participant had a university degree or beyond. The participants reported earning an annual farm income of Uganda shillings (UGX) 1,668,493, equivalent to USD 461.62 or 392.08 Euros. The respondents had on average 4.2 y of experience growing improved crop varieties, but only 37.8% of them had heard about biotechnology in agriculture. When asked how often they have been growing improved crop varieties, 39.8% of the respondents reported that they have never grown improved crop varieties, while 10.9% indicated always having cultivated improved crops varieties every planting season for the past 2 y. Overall, farmers exhibited positive adoption intentions (Mean = 4.56, Standard deviation = 0.54) for GE cassava.

**Table 2.** Sociodemographic characteristics of the study sample consisting of cassava growers in Gulu district, Northern Uganda (*n* = 357).

Respondent characteristics	Frequency	Percentage (%)	Respondent characteristics	Mean	Standard deviation
**Gender**			Age (years)	42.3	33.12
Male	138	38.7	Household size	6.29	2.91
Female	219	61.3	Average annual income (UGX)	1,668,493	1,642,339
**Education**			Experience with improved crop varieties (years)	4.21	5.42
No formal education	37	10.4	Farm size (acres)	4.87	2.94
Primary education	237	66.4	Cassava growing area (acres)	1.46	2.64
Secondary education	69	19.3	Farming experience (years)	20.17	13.81
^a^Other institutions	14	3.9			
**Awareness of biotechnology**					
Yes	135	37.8			
No	222	62.2			

^a^Other institutions refer to tertiary institutions including vocational schools but not universities.

### Measurement Model

The reflective measurement model results are presented in Table 3. Cronbach’s alpha and composite reliability values of all constructs exceeded the recommended value of 0.7,^107,108^ revealing a satisfactory consistency and construct reliability of the proposed measurement model. Similarly, the convergent validity scores, given by the AVE values for each measured construct, were above the recommended threshold of 0.5.^107^

**Table 3.** Reliability and validity statistics.

Construct	Cronbach’s alpha	Composite reliability	Average variance extracted (AVE)
ATT	0.89	0.92	0.69
NAE	0.93	0.95	0.87
PAE	0.84	0.90	0.76
PBC	0.73	0.84	0.63
SN	0.82	0.88	0.65
DE	0.76	0.89	0.81
AI	0.93	0.96	0.88

Note: ATT = attitude, NAE = negative anticipated emotions, DE = desire, PAE = positive anticipated emotions, PBC = perceived behavioural control, SN = subjective norms, AI = adoption intentions.

Regarding the Variance Inflation Factor (VIF), all values were below the threshold of 5, demonstrating the absence of multicollinearity within the respective construct items. Also, an assessment of the discriminant validity revealed that all HTMT values (Table 4) did not exceed the recommended value of 0.85,^109^ except for the value for DE/AI which was 0.897, but is still below the threshold of 0.90 as suggested by Henseler et al.^111^ This means that all constructs included in the model were distinct from each other.

**Table 4.** Discriminant validity of the model based on the Heterotrait-Monotrait ratio (HTMT).

	AI	ATT	DE	NAE	PAE	PBC	SN
AI							
ATT	0.721						
DE	0.897	0.741					
NAE	0.042	0.03	0.084				
PAE	0.663	0.562	0.701	0.121			
PBC	0.648	0.617	0.688	0.108	0.67		
SN	0.669	0.767	0.618	0.052	0.584	0.587	

Note: ATT = attitude, NAE = negative anticipated emotions, DE = desire, PAE = positive anticipated emotions, PBC = perceived behavioural control, SN = subjective norms, AI = adoption intentions.

### Structural Model

The path coefficients, effect sizes (f_2_), corresponding *p*-values and significance levels of the hypothesized relationships are presented in Table 5. The adoption intention (AI) for GE CBSD-resistant cassava was positively and significantly influenced by DE (β = 0.497, *p* < .05), PBC (β = 0.106, *p* < .001), ATT (β = 0.165, *p* < .05) and SN (β = 0.19, *p* < .05), with DE having the largest effect on AI (f^2^ = 0.395). As hypothesized, PAE (β = 0.263, *p* < .05), ATT (β = 0.346, *p* < .05) and PBC (β = 0.182, *p* < .05) also had significant positive influences on DE. In contrast, SN and NAE had no significant influence on DE.

**Table 5.** Results from analysis of path coefficients.

Hypothesis	Path	Path coefficient	*p*-values	f-squared	Hypothesis conclusion
	***Direct effects***				
H1	PAE ->DE	0.263**	0.001	0.087	Accept
H2	NAE ->DE	0.027	0.521	0.001	Reject
H3a	ATT ->DE	0.346**	0.009	0.116	Accept
H3b	ATT ->AI	0.165**	0.026	0.035	Accept
H4a	SN ->DE	0.079	0.422	0.005	Reject
H4b	SN ->AI	0.19**	0.015	0.054	Accept
H5a	PBC ->DE	0.182**	0.003	0.041	Accept
H5b	PBC ->AI	0.106***	0.000	0.021	Accept
H6	DE ->AI	0.497***	0.000	0.395	Accept
	***Indirect effects***				
	NAE ->DE ->AI	0.013			
	PAE ->DE ->AI	0.131**			
	PBC ->DE ->AI	0.091**			
	SN ->DE ->AI	0.037			
	ATT ->DE ->AI	0.172**			

Note: ATT = attitude, NAE = negative anticipated emotions, DE = desire, PAE = positive anticipated emotions, PBC = perceived behavioural control, SN = subjective norms, AI = adoption intentions, ** significance level of 0.05, *** significance level of 0.001.

Accordingly, all indirect effects of the independent constructs on AI, mediated by desire, were found to be significant and pointing in the same direction as the direct effects, except for SN and NAE that had no significant indirect effects on AI. Desire partially mediated the effects of ATT and PBC on AI, while showing no mediation for the effect of SN on AI (see indirect effects in Table 5). This underscores the importance of desire as a motivation for AI toward agricultural technologies, and emphasizes the role of indirect effects in reinforcing the magnitude and direction of the direct effects.^99^

Using the blindfolding procedure, we obtained the Stone-Geisser’s Q^2^ values to show the predictive relevance of the structural model.^107^ All the endogenous constructs had medium and high predictive relevance for desire and AI, respectively (Q^2^ _DE = 0.446, Q^2^ _AI = 0.515). The percentage of extracted variance, R^2^ values for DE and AI were 50.5% and 66.3%, respectively.

Based on both Henseler’s^114^ and permutation^115^ MGA methods, the results of the PLS-MGA reveal significant differences between respondents with low and high education with respect to the influence of ATT on AI and between lowly and highly experienced farmers for the PAE -> DE path. Accordingly, the influence of ATT on AI and PAE on DE are much higher for farmers with low education and farming experience compared to their respective counterparts. However, for all the other path coefficients and relationships across all groups, the MGA results show no significant differences. Both MGA methods showed consistent significant/nonsignificant differences in the structural path (Appendix 1) between respective groups, thus proving the credibility of our results based on this multi-method confirmation.^116^

## Discussion

This study explored the ex-ante adoption intentions of Ugandan farmers for GE cassava and, in doing so, it brings the perspectives of African farmers into the GE debate, contributing to the growing body of research on GE technologies in agriculture. Specifically, it examined farmers’ adoption intentions for GE CBSD-resistant cassava within a hypothetical context in Northern Uganda, where respondents have no prior experience with GE crops. The findings therefore capture farmers’ responses based on exposure to an information script, rather than real-world adoption behavior.

Overall, the PLS model used in this study explained most of the variance in farmers’ AI toward GE cassava. While not all the constructs showed statistical significance, our findings are consistent with previous literature and confirm that both affective and cognitive factors are important determinants of adoption intentions toward novel agricultural technologies.^73–75^

Consistent with the MGB, ATT and PBC emerged as significant predictors of both desire and adoption intentions. This accentuates the idea that farmers’ evaluative beliefs and self-efficacy are pivotal in shaping their intentions to adopt a technology. In contrast, SN did not significantly influence desire, but had a direct effect on AI. This finding compares with Langer et al.^74^ and Arora et al.^117^ who found no effect of SN on desire of farmers to use IoT sensors on their dairy cattle and consumers’ intent behind webrooming, respectively. However, it contrasts with earlier research that reported a significant influence of SN on desire.^73,84^ This indicates that desire, though posited as a strong proximal mediator in the MGB, may not uniformly mediate the effects of all TPB constructs on AI.^60^ The divergence in the effects of SN on desire and AI reveals an important nuance. While social influence remains relevant, it appears to operate primarily through a compliance-oriented pathway rather than through internalized motivation. In other words, farmers’ social networks may not necessarily generate an intrinsic desire to cultivate GE cassava, but rather enhance their intentions through compliance to their networks. This finding is particularly salient in contexts where autonomy and collectivism co-exist, which is typical of many developing country settings. It further reechos previous research with Ugandan farmers demonstrating that farmer associations, extension visits and prior experience strongly condition intentions to adopt novel biotech crops.^52^ Furthermore, this study refines the application of norm-inclusive models such as the MGB in sub-Saharan African contexts. Specifically, it demonstrates that normative influences can shape behavioral intentions without necessarily translating into goal-directed desire, particularly under conditions of technological novelty and uncertainty. This challenges the assumption of uniform mediation by desire^43^ and suggest that the motivational architecture of adoption decisions may be more context dependent than previously recognized in research. From an agricultural policy and extension standpoint, this suggests that interventions should leverage social networks as channels of peer influence rather than as drivers of intrinsic motivation. For instance, farmer groups, co-operatives and influential individuals in society can be strategically engaged to legitimize GE technology and reinforce its social acceptability, even where individual intrinsic motivation remains limited.

Consistent with the MGB assumptions, desire exerted the strongest and statistically significant influence on AI. Moreover, it partially mediated the effects of ATT and PBC on AI as posited by Perugini and Bagozzi.^43^ This corroborates earlier findings that position desire as an important motivational driver for decision making.^73,91^ The prominent effect of desire in this model could also mean that beyond the cognitive factors on which farmers mostly base their AI, they also align the novel technologies with their personal goals, which in this case could be to cultivate CBSD-resistant cassava, obtain higher yields or improve their farm income. This highlights the importance of integrating farmers’ farming priorities when developing such technologies. Practically, this implies that breeding programs and extension services should explicitly align GE traits with farmers’ priorities such as yield stability, disease resistance and improved income, and communicate these benefits in a goal-oriented and locally meaningful manner. Framing GE crops in terms of tangible livelihood outcomes is likely to strengthen farmers’ desire to adopt these crops, and in turn, their adoption intentions.

Regarding emotions, PAE significantly influenced desire to adopt GE cassava, whereas NAE did not. This asymmetric pattern aligns with recent empirical and meta-analytic evidence,^67,118^ and fits with global genome editing evidence showing that acceptance rises when benefits are concrete and value aligned. Moreover, positive emotions are often associated with more positive intentions to perform a behavior,^75^ implying that farmers are more likely to develop stronger desire and ultimately intention to adopt GE cassava, if they anticipate that it will positively contribute to the achievement of their farming goals. The insignificant effect of the NAE on desire may reflect the relatively weak emotional salience of expected negative outcomes in the context of this study. As suggested by Perugini and Bagozzi,^43^ anticipated emotions may only exert meaningful influence on desire when they are strongly felt. In this study, the attenuated effect of NAE could be partly attributed to the novel nature of GE crops, the hypothetical context of this study, and the fact that farmers have no experience with GE crops. Previous research shows that emotional responses to technologies are closely linked to familiarity and prior exposure^119^ and that farmers’ adoption decisions for agricultural innovations are often shaped by their prior experiences with similar technologies.^120^ Consequently, in ex-ante contexts, such as for this study, emotional responses may remain underdeveloped due to the absence of experience-based emotional associations with the technology. This is further supported by contrasting evidence from contexts where technology users have prior experience with a technology. For instance, Landmann et al.^73^ found a significant effect of NAE on Indian farmers’ desire to use smart phones, a technology respondents already had experience with. This further underscores how emotional influence depends on experience and technological familiarity, which remain limited in the case of GE crops in Uganda. Practically, these findings suggest that early-stage communication strategies should prioritize benefit-oriented messaging while simultaneously considering experiential approaches for the introduction of the GE crops. Accordingly, demonstration plots and farmer field schools could help translate the abstract benefits into tangible experiences, thereby strengthening PAE. Concurrently, transparent and proactive communication regarding potential risks remains essential to ensure that farmers’ adoption intentions are shaped through credible and balanced information rather than misinformation or uncertainty.

Results of the PLS-MGA revealed variation in the moderation effect of education and farming experience on the paths ATT -> AI and PAE -> DE, respectively. The significant ATT -> AI relationship among low educated farmers suggests their reliance on evaluative judgment when forming their adoption intention. Whereby, their attitude toward technology may serve as a primary determinant for their behavioral intentions. In contrast, for more highly educated farmers, the relationship appears weaker, which may reflect their ability to incorporate a broader set of considerations such as more detailed assessment of potential risks and benefits, and long term implications of adopting GE crops when forming their adoption intentions. Similarly, the stronger PAE -> DE path among less experienced farmers indicates that positive emotions more strongly stimulate motivational desire when experiential knowledge is limited. In the absence of farming experience with similar technologies, farmers may rely more on affective cues to guide their decision making. This is consistent with evidence that individuals depend more on emotion-based heuristics under conditions of limited knowledge or uncertainty.^121^ In contrast, more experienced farmers may draw on prior knowledge and practical experience to evaluate the potential outcome of adopting the novel GE cassava, instead of relying on their anticipated emotions. Moreover, previous research on GM crop adoption suggests that farmers’ prior experience and exposure to agricultural innovations play an important role in shaping adoption behavior and the formation of perceptions and expectations regarding new technologies.^122^

The findings of this study show heterogeneity in adoption intention pathways among farmers, where certain paths do not operate uniformly across socio-demographic groups. This implies that a “one size fits all” approach to promoting GE technologies may not be effective. Rather, extension strategies should be tailored to specific farmer segments. For instance, more educated and experienced farmers may require more detailed, evidence-based information including risk-benefit analyses and comparative field results, while less educated and less experienced farmers may benefit more from simplified, visually supported communication with clear and relatable messaging to strengthen their attitude, and experiential learning approaches such as demonstration trials and participatory approaches to enhance their goal-oriented motivations.

In summary, findings of this study entail important implications for key stakeholders involved in the development and dissemination of GE crops. For breeders, intentions for technology adoption could be improved by aligning the GE traits with farmers’ personal goals and farming realities. This underscores the importance of demand-driven and context-specific trait development. For extension agents and science communicators, the findings highlight the need for targeted and experience-based strategies that translate anticipated benefits into tangible outcomes, for instance through demonstration plots and farmer field schools. While strengthening farmers ATT and PBC remains critical for fostering motivation and intention, the continued influence of SN on AI suggests that community-based approaches such as peer learning platforms and engagement with trusted local actors remain highly relevant. Overall, these findings highlight the need for differentiated, context-sensitive intervention strategies, while recognizing that pathways to adoption intention formation are heterogeneous.

Importantly, interpretation of the findings of this study should also take into account the specific study contexts of Northern Uganda, considering that farmers’ adoption intentions and perceptions may differ in other regions of the country with varying agro-ecological conditions, market and institutional environments.^123,124^ For instance, farmers operating in regions with low CBSD pressure or more diversified cropping systems may evaluate the GE cassava differently, hence generalization of this study’s findings should be done with caution.

### Limitations

Several limitations should be considered when interpreting the results of this study. First, the study examines farmers’ adoption intentions in a hypothetical context. Respondents had no prior exposure to GE crops, and their responses were solely based on the information provided to them during the survey. This raises the possibility of hypothetical bias, whereby respondents may overstate their adoption intentions, compared to their actual behavior under real-world conditions characterized by economic trade-offs, agronomic and institutional constraints, and uncertainties.^125,126^ Future research could address this limitation by examining adoption intentions in contexts where actual GE crops are commercially available and further test whether intentions eventually translate into actual adoption in the long run.

Second, while the information script provided was intended to provide a basic understanding of GE to the respondents, the study did not formally test the understanding of technical concepts such as DNA, GMOs and GE by the respondents. Instead, comprehension was inferred from enumerator explanations and respondents’ verbal confirmation. While this approach is practical in field settings, especially during face-to-face interviews, future studies could consider incorporating structured comprehension checks prior to data collection, particularly in rural smallholder contexts where familiarity with such concepts may be limited. In addition, the use of locally relevant analogies such as comparisons of GE with conventional breeding techniques, and explicit reference to local regulatory positions of respective study contexts could enhance comprehension and provide a more contextually grounded basis for evaluation.

Third, our study only focused on negative and positive anticipated emotions as affective drivers of intentions. This is general and may not give a comprehensive understanding of affective influences on intentions. Future studies linking affective factors to farmers’ behavioral intentions could consider including constructs such as trust, perceived fairness and moral concerns in the model. Furthermore, integrating economic variables such as expected profitability, input costs and credit access into the MGB would enable a more comprehensive understanding of adoption decisions among farmers, allowing for a more holistic analysis of determinants combining behavioral drivers with economic factors that are fundamental in shaping actual adoption behavior.

Fourth, the use of purposive sampling of cassava farmers within a single district limits the generalizability of the findings. While the study area was selected as a highly relevant context characterized by strong dependence on cassava and high CBSD prevalence, farmers’ evaluations may still vary across regions and value chains. This variation can be due to several factors including consumption habits, varietal trait preferences and specific agroecological and institutional constraints.^15,17^ As such, the results should be interpreted as context-specific insights rather than generalizable evidence. Future research could address this limitation by expanding the geographical scope and including farmers from different value chains to enable comparative analysis across diverse farming contexts. This would allow for a more nuanced understanding of how contextual factors influence adoption intentions for GE, and further enhance the external validity and generalizability of findings across heterogeneous agricultural settings.

Finally, as with most survey-based research, responses may be subject to social desirability bias.^127^ This is evident in the overall high adoption intentions, which could be a reflection of bias rather than grounded adoption intentions. Primarily, this could be due to respondents expressing positive intentions with expectations of gaining access to the CBSD-resistant cassava or their comprehension of the information script provided. This may compromise the construct validity of the measures of adoption intentions, as responses may partly reflect perceived expectations rather than true adoption intention. Consequently, the reported adoption intention should be interpreted with caution. To address this limitation, future research could employ more experimental or scenario-based approaches to study farmers’ adoption decisions in real world situations, for instance, by experimentally varying attitude-based benefit information or emotion eliciting scenarios to assess their effects on desire and adoption intentions.

## Conclusions

The findings of this study demonstrate that both affective and cognitive factors play an important role in shaping farmers’ motivations and adoption intentions for emerging agricultural technologies. In the case of GE cassava resistant to CBSD in Uganda, PAE, ATT and PBC were particularly influential, underscoring the importance of dual process perspectives in agricultural technology adoption literature. Desire emerged as a strong predictor of farmers’ AI, emphasizing its theoretical relevance in goal directed decision making processes, and reinforcing the importance of aligning technology development with farmers’ production goals. The insignificant effect of SN on desire, and its significant direct effect on intention offers an interesting insight into the role of normative compliance in shaping intentions directly rather than through motivational pathways. The asymmetrical influence of emotions on desire, and consequently intentions, where only PAE had an influence on desire highlights that emotional responses toward novel technologies like GE are shaped by anticipated benefits and alignment with farmers’ production goals. Therefore, in designing implementation strategies for GE crop cultivation with farmers, it is important to consider their personal goals and emphasize the benefits anticipated from its cultivation. In sum, this study extends the theoretical application of the MGB to agricultural biotechnology and points out practical strategies for future deployment of GE crops. Leveraging on positive attitudes, enhancing farmers’ perceived control and aligning the GE attributes with farmers’ goals is critical. Moreover, scientists and extension agents could integrate these insights into farmer trainings, communications and participatory engagement of end-users to support a more informed, context-appropriate introduction of GE crops in Uganda.

## Supplementary Material

Supplementary Material.docx

## Appendix

**Table A1.** Results of the PLS-MGA.

	Path coefficients			p-value (2-tailed)		
Relationships	Low education, N = 274	High education, N = 83	Path coefficient difference	Henseler’s MGA	Permutation MGA	Significant?
ATT -> AI	0.269**	−0.175	0.444*	0.006	0.008	Yes/yes
ATT -> DE	0.370*	0.302*	0.067	0.694	0.937	No/No
DE -> AI	0.453**	0.567**	−0.114	0.429	0.515	No/No
NAE -> DE	0.037	0.015	0.022	0.757	0.809	No/No
PAE -> DE	0.217*	0.429*	−0.212	0.203	0.302	No/No
PBC -> AI	0.085	0.191	−0.106	0.360	0.378	No/No
PBC -> DE	0.232**	−0.030	0.263	0.102	0.074	No/No
SN -> AI	0.180*	0.298*	−0.118	0.448	0.564	No/No
SN -> DE	0.085	0.052	0.034	0.887	0.921	No/No
	**Young, *N* = 150**	**Old, *N* = 207**				
ATT -> AI	0.066	0.249*	−0.183	0.196	0.227	No/No
ATT -> DE	0.059	0.462*	−0.403	0.068	0.230	No/No
DE -> AI	0.520**	0.427**	0.093	0.460	0.471	No/No
NAE -> DE	0.113	0.045	0.068	0.477	0.390	No/No
PAE -> DE	0.385**	0.149	0.235	0.122	0.169	No/No
PBC -> AI	0.134	0.080	0.054	0.558	0.589	No/No
PBC -> DE	0.121	0.191*	−0.070	0.539	0.603	No/No
SN -> AI	0.218	0.217*	0.001	0.965	0.994	No/No
SN -> DE	0.274	0.091	0.183	0.323	0.428	No/No
	**Low income, *N* = 222**	**High income, *N* = 135**				
ATT -> AI	0.180*	0.033	0.147	0.440	0.351	No/No
ATT -> DE	0.247	0.566*	−0.319	0.276	0.375	No/No
DE -> AI	0.488**	0.616**	−0.127	0.443	0.431	No/No
NAE -> DE	0.074	−0.011	0.085	0.214	0.271	No/No
PAE -> DE	0.255*	0.229	0.026	0.852	0.889	No/No
PBC -> AI	0.108	0.078	0.030	0.779	0.775	No/No
PBC -> DE	0.115	0.315*	−0.200	0.149	0.119	No/No
SN -> AI	0.183*	0.227	−0.044	0.961	0.805	No/No
SN -> DE	0.151	−0.134	0.284	0.284	0.213	No/No
	**Low farming experience, *N* = 207**	**High farming experience, *N* = 150**				
ATT -> AI	0.099	0.278*	−0.179	0.199	0.256	No/No
ATT -> DE	0.072	0.492*	−0.421	0.060	0.204	No/No
DE -> AI	0.562**	0.349**	0.213	0.102	0.140	No/No
NAE -> DE	0.038	0.036	0.002	0.907	0.978	No/No
PAE -> DE	0.406**	0.078	0.327*	0.049	0.049	Yes/yes
PBC -> AI	0.088	0.123	−0.035	0.713	0.710	No/No
PBC -> DE	0.114	0.223*	−0.109	0.343	0.387	No/No
SN -> AI	0.191*	0.237*	−0.046	0.794	0.782	No/No
SN -> DE	0.268*	0.109	0.159	0.283	0.520	No/No
	**Small farm size, *N* = 252**	**Large farm size, *N* = 105**				
ATT -> AI	0.206*	−0.015	0.222	0.118	0.208	No/No
ATT -> DE	0.353*	0.320*	0.032	0.803	0.941	No/No
DE -> AI	0.491**	0.515**	−0.024	0.888	0.887	No/No
NAE -> DE	0.042	−0.010	0.052	0.437	0.512	No/No
PAE -> DE	0.263*	0.273*	−0.010	0.912	0.965	No/No
PBC -> AI	0.145*	0.040	0.105	0.253	0.326	No/No
PBC -> DE	0.232*	0.053	0.178	0.165	0.221	No/No
SN -> AI	0.118	0.393*	−0.275	0.100	0.110	No/No
SN -> DE	−0.005	0.295*	−0.299	0.091	0.219	No/No

Note: ATT = attitude, NAE = negative anticipated emotions, DE = desire, PAE = positive anticipated emotions, PBC = perceived behavioral control, SN = subjective norms, AI = adoption intentions; Young farmers =<35 y, Low education =<primary education, Low income =<UGX 1,500,000, Low farm experience =<20 y, Small farm size =<4.5 acres; ** significance level of 0.05, *** significance level of 0.001.
