# Supplementary material for: Drivers of farmers’ intentions to adopt genome-edited cassava resistant to cassava brown streak disease in Uganda
Source: GM Crops Food. 2026 Jul 8;17(1):2698267. doi: 10.1080/21645698.2026.2698267 (PMC13353787; doi:10.1080/21645698.2026.2698267)
Supplement: Supplementary Material.docx [file KGMC_A_2698267_SM4275.docx]

**Supplementary material 1: Information script**

**Description of Genome editing**

All living things have DNA, which contains instructions needed for them to grow, develop and reproduce; more like an instruction manual. Genome editing is a technique that allows scientists to make small, precise changes to this DNA in plants, animals, and even humans. Imagine it as carefully correcting a few words or letters in a book without rewriting the whole book. In agriculture, genome editing is being used to help crops resist diseases (e.g. cassava varieties resistant to the Cassava Brown Streak Disease, Bananas resistant to bacterial wilt and sorghum tolerant to striga weed), increase their resilience to drought; prolong their shelf life and improve their nutrient content, etc. Unlike GMOs, genome editing usually works within the plant’s own DNA, without involving addition of foreign DNA from other organisms. Because genome editing technology is still new, there is ongoing discussions regarding its benefits, potential risks, and long-term impacts in many parts of the world. Questions still remain on how genome editing may affect the environment, health, and whether smallholder farmers will have access to genome edited seeds. As a farmer, you are a direct stakeholder in the implementation of this technology, so your voice matters in ensuring the technology supports your needs and priorities.

**Supplementary table 1: MGB constructs and measurement items**

| **Constructs** | **Items and Sources** |
| --- | --- |
| **Attitude** | (Ajzen, 2006; Perugini & Bagozzi, 2001) |
| ATT1 | It would be a good idea for me to cultivate GE cassava |
| ATT2 | Adopting GE cassava would enhance my farm’s productivity |
| ATT3 | I do not like the idea of cultivating GE cassava on my farm |
| ATT4 | I am positive towards cultivating GE cassava on my farm |
| AT5 | Growing GE cassava could be beneficial for my farm |
| **Subjective Norms** | (Ajzen, 2006; Langer et al., 2024) |
| SN1 | People who are important to me would adopt GE cassava |
| SN2 | Religious leaders and cultural leaders not support my decision to grow GE cassava |
| SN3 | People who are important to me would think that I should cultivate GE cassava |
| SN4 | My fellow farmers would support the use of GE cassava |
| **Perceived Behavioural Control** | (Ajzen, 2006) |
| PBC1 | I am confident in my ability to grow GE cassava if made available |
| PBC2 | If I want to, I would grow GE cassava |
| PBC3 | It is up to me to decide whether to cultivate GE cassava on my farm or not |
| **Positive Anticipated Emotions** | (Landmann et al., 2020; Perugini & Bagozzi, 2001) |
| PAE1 | I would feel proud if I successfully grow GE cassava |
| PAE2 | I would feel satisfied if I successfully grow GE cassava |
| PA3 | I would feel happy if GE cassava performs well in my garden |
| **Negative Anticipated Emotions** | (Landmann et al., 2020; Langer et al., 2024; Perugini & Bagozzi, 2001) |
| NAE1 | I would feel angry if GE cassava does not meet my expectation |
| NAE2 | I would feel disappointed if GE cassava does not meet my expectation |
| NAE3 | I would feel sad if GE cassava does not meet my expectation |
| **Desire** | (Landmann et al., 2020; Langer et al., 2024; Perugini & Bagozzi, 2001) |
| DE1 | I desire to grow GE cassava if it is available |
| DE2 | I want to cultivate GE cassava to improve cassava production on my farm |
| **Adoption intentions** | (Ajzen, 2006; Zobeidi et al., 2022) |
| AI1 | I plan to acquire GE cassava when they become available |
| AI2 | I will make an effort to integrate GE cassava into my farming practices |
| AI3 | I intend to grow GE cassava as soon as it becomes available |

**References**

Ajzen, I. (2006). Constructing a theory of planned behavior questionnaire. In: Amherst, MA.

Landmann, D., Lagerkvist, C.-J., & Otter, V. (2020). Determinants of Small-Scale Farmers’ Intention to Use Smartphones for Generating Agricultural Knowledge in Developing Countries: Evidence from Rural India. *The European Journal of Development Research*, *33*(6), 1435-1454. <https://doi.org/10.1057/s41287-020-00284-x>

Langer, G., Schulze, H., & Kühl, S. (2024). From intentions to adoption: Investigating the attitudinal and emotional factors that drive IoT sensor use among dairy farmers. *Smart Agricultural Technology*, *7*. <https://doi.org/10.1016/j.atech.2024.100404>

Perugini, M., & Bagozzi, R. P. (2001). The role of desires and anticipated emotions in goal-directed behaviours: broadening and deepening the theory of planned behaviour. *Br J Soc Psychol*, *40*(Pt 1), 79-98. <https://doi.org/10.1348/014466601164704>

Zobeidi, T., Yaghoubi, J., & Yazdanpanah, M. (2022). Exploring the motivational roots of farmers' adaptation to climate change‑induced water stress through incentives or norms. *Scientific Reports*, *12*(1), 15208. <https://doi.org/10.1038/s41598-022-19384-1>
